# Stimulus Phase Locking of Cortical Oscillation for Auditory Stream Segregation in Rats

**DOI:** 10.1371/journal.pone.0083544

**Published:** 2013-12-20

**Authors:** Takahiro Noda, Ryohei Kanzaki, Hirokazu Takahashi

**Affiliations:** 1 Research Center for Advanced Science and Technology, The University of Tokyo, Tokyo, Japan; 2 Department of Mechano-Informatics, Graduate School of Information Science and Technology, The University of Tokyo, Tokyo, Japan; 3 Precursory Research for Embryonic Science and Technology, Japan Science and Technology Agency, Saitama, Japan; Instituto de Neurociencias de Alicante UMH-CSIC, Spain

## Abstract

The phase of cortical oscillations contains rich information and is valuable for encoding sound stimuli. Here we hypothesized that oscillatory phase modulation, instead of amplitude modulation, is a neural correlate of auditory streaming. Our behavioral evaluation provided compelling evidences for the first time that rats are able to organize auditory stream. Local field potentials (LFPs) were investigated in the cortical layer IV or deeper in the primary auditory cortex of anesthetized rats. In response to ABA- sequences with different inter-tone intervals and frequency differences, neurometric functions were characterized with phase locking as well as the band-specific amplitude evoked by test tones. Our results demonstrated that under large frequency differences and short inter-tone intervals, the neurometric function based on stimulus phase locking in higher frequency bands, particularly the gamma band, could better describe van Noorden’s perceptual boundary than the LFP amplitude. Furthermore, the gamma-band neurometric function showed a build-up-like effect within around 3 seconds from sequence onset. These findings suggest that phase locking and amplitude have different roles in neural computation, and support our hypothesis that temporal modulation of cortical oscillations should be considered to be neurophysiological mechanisms of auditory streaming, in addition to forward suppression, tonotopic separation, and multi-second adaptation.

## Introduction

**Figure 1:**
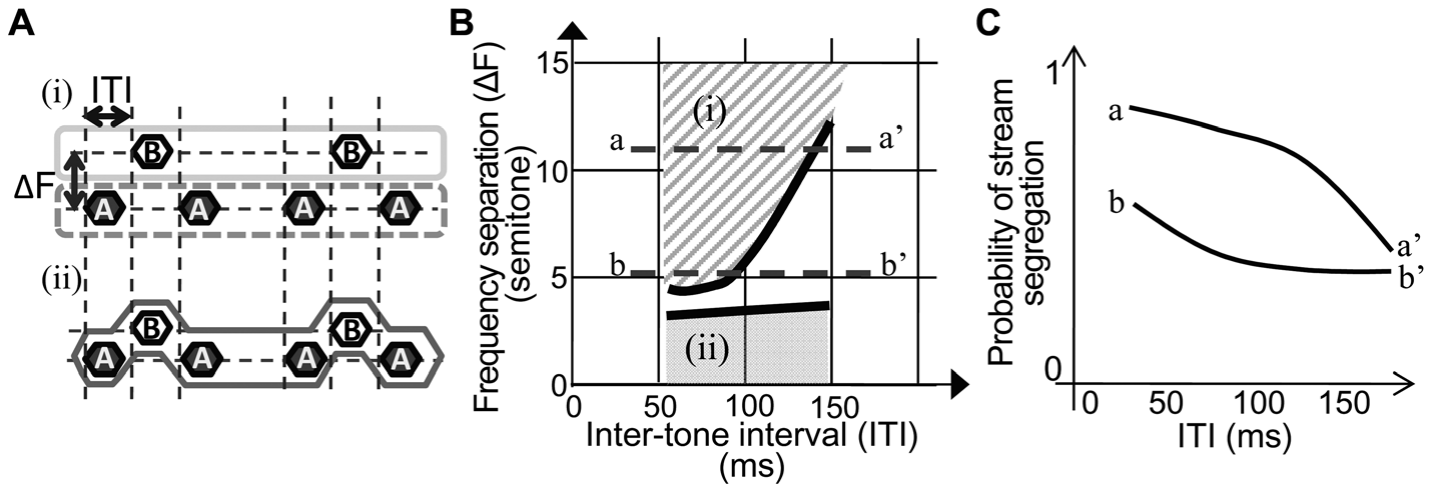
Auditory stream segregation of an alternating tone sequence. **A.** Perception of the ABA- sequence. Depending on ITI and ΔF, ABA- sequence induces either segregated perception of A-tone and B-tone sequences (i) or integrated perception of the ABA- sequence (ii). **B.** The psychometric function in the ITI–ΔF plane (van Noorden, 1975). Segregated perception is induced in zone (i), whereas integrated perception is induced in zone (ii). The lower limit of segregated perception zone and the upper limit of integrated perception zone are the “temporal coherence boundary” and “fission boundary,” respectively. **C.** Putative probability of stream segregation at indicated cross-sections in the psychometric function **B.**

The auditory neural system can extract and reconstruct coherent perceptual objects from the complex acoustic inputs in daily environments. In laboratory experiments, perceptual integration and segregation based on the time–frequency properties of artificial sounds is known as auditory streaming. For example, an alternating tone sequence differing in frequency (ABA-ABA-…) induces different streams depending on the differences in frequency (ΔFs) between tones A and B and the inter-tone intervals (ITIs) between successive tones (Fig. 1 **A** and **B**) [1]. Tone sequences with larger ΔFs and shorter ITIs are more likely to be perceived as segregated streams of A-A-A- and B---B---, whereas sequences with smaller ΔF are more likely to be perceived as integrated streams of ABA-ABA-, irrespective of ITI. At intermediate ΔFs and longer ITIs, however, perception varies from trial to trial.

Until date, tonotopic separation and forward suppression in the auditory cortex have been considered as neural mechanisms of van Noorden’s perceptual boundary [2], [3]. Because of spatial separation on the tonotopic map, neural populations responsive to tones A and B become more distinct with increasing ΔF, thereby weakening the forward suppression in these populations. This renders the interaction between these tones less effective, leading to the perception of segregated streams. In contrast, this inter-tone interaction is enhanced with decreased ΔF, leading to the perception of an integrated stream. Furthermore, multi-second adaptation of auditory cortical response may also contribute to the build-up of streaming over time [4]. In the temporal domain, however, the forward suppression may not completely account for van Noorden’s perceptual boundary predicting that a shorter ITI is more likely to induce segregated stream perception (Fig. 1 **C**): shortening ITI enhances the inter-tone interaction [5]–[7], by which the forward suppression by B tone to the subsequent A tone increases; consequently, the last A tones in the triplet evoke smaller responses than the first A tones [2], [4], [8], [9], contradicting the perception that the first and last A tones are indistinguishable in a segregated stream of A-A-A- sequence.

In addition to these mechanisms, the build-up of streaming over seconds may be due to the long-time-scale evolution of specific cortical states. In the visual cortex, evolution of oscillatory ongoing activities is closely linked with temporally organized discharges or synchronization of distinct neural populations encoding a coherent object [10]. These cortical oscillatory activities are modulated in varied computations ranging from sensorimotor to higher-order cognitive functions by both top-down task-specific attentions and bottom-up stimulus properties [11]–[13]. In the formation of an auditory stream, a limited volitional control implies important roles of bottom-up, stimulus-driven modulation [14], possibly in terms of oscillation; such bottom-up streaming is referred to as a ‘primitive’ streaming.

Considering the rapid decrease in the magnitude of the cortical response to stimulus sequences [15]–[17], oscillation amplitude may not explain the build-up effect, which usually requires several seconds. On the other hand, a stimulus-dependent oscillatory phase may serve as a gate of inputs, which underlies the formation and disruption of stimulus perception [18], [19]. The inputs with a ‘proper’ phase lead to signal amplification in the cortex, whereas those with an ‘improper’ phase lead to suppression [20]. Furthermore, as periodic inputs lead to a reproducible phase pattern, the phase reliability or phase coherence may also contribute to prediction of ongoing stimuli, which is crucial to perception [21], [22]. Thus, the stimulus-dependent oscillatory phase is considered as a possible neural correlate of stream perception [21]–[23].

Here we test the hypothesis that oscillatory phase modulation, instead of amplitude modulation, is a neural correlate of auditory streaming, which should satisfy the following two conditions: (i) the neurometric property correlates with van Noorden’s perceptual boundary at short ITI; and (ii) the neurometric property builds up over time. In the present study, we first provide an evidence of auditory streaming in rodents through behavioral experiments. A lack of evidence of auditory streaming in animal models, except for a monkey, ferret and song bird, has limited investigation of the neural correlates of auditory streaming to date. Evidence of auditory streaming in other species would thus be encouraging and help advance in this field. Second, in the primary auditory cortex of anesthetized rats, we comprehensively investigated the band-specific phase locking as well as the amplitude of stimulus-locked oscillatory activities of LFPs when manipulating ΔF and ITI of alternating tone sequences. These series of experiments demonstrate that the oscillatory phase modulation plays a crucial role in auditory streaming.

## Materials and Methods

This study was conducted in strict accordance with “Guiding Principles for the Care and Use of Animals in the Field of Physiological Science” published by the Japanese Physiological Society. The experimental protocol was approved by the Committee on the Ethics of Animal Experiments at the Research Center for Advanced Science and Technology, the University of Tokyo (Permit Number: RAC09107). All the surgeries were performed under isoflurane anesthesia, and every effort was made to minimize suffering.

### Behavioral experiment

**Animal preparation.** Four adult male Wistar rats were used in the experiments. On the first day of training, their postnatal week was 11 and body weight was approximately 320 g. These rats had no prior experience of operant conditioning procedures. The rats had restricted access to dry food, but free access to water all the time. Animal condition was carefully monitored on a daily basis. Their weights were maintained at 80–85% of ad libitum.

**Appratus.** Behavioral experiments were conducted in a custom-made operant chamber with a dimension of 34.5×34.5×34.5 cm^3^ (OPFZ-3001; O’hara & Co. Ltd., Tokyo, Japan), which was placed in a soundproof booth (80×70×70 cm^3^) (Japan Shield Enclosure Co. Ltd., Osaka, Japan). The chamber was equipped with two 15-mm-diameter poking holes (left and right holes) on the wall in a front panel and a food dispenser in a rear panel. The poking holes were covered by a sliding door, which restricted accesses to the holes. A green-colored LED was attached in the back of each poking hole to provide cues according to experimental procedures. A free field speaker (EAS-10TH800; Panasonic Corp., Osaka, Japan) was placed at 35 cm above the arena. A LED array placed on the ceiling of soundproof booth illuminated the arena, keeping the illuminance at 50 lux.

**Stimuli.** Four kinds of test stimulus sequences [24] were used in the behavioral experiments as summarized in Table 1; the slow isochronous (frequency, 20 – 40.2 kHz; ITI, 400 ms), fast isochronous (20 kHz; 200 ms), galloping (20 – 40.2 kHz; 100 ms) and ABA- sequences (A-tone frequency, 20 kHz; B-tone frequency, 22.5 – 40.2 kHz; ITI, 100 ms). Each sequence consisted of tone bursts with 70 dB SPL, 30 ms duration, and 5 ms linear rise/fall ramps. The SPL calibration of test stimuli were conducted by a microphone (Brüel & Kjær, 4939) placed at the center of arena at the height of 15 cm, where the distance from the speaker was 30 cm.

**Table 1:** Test stimulus used in behavioral experiments.

Test stimulus sequence	ITI (ms)	Stimulus freq. (kHz)	Notes
(i) Slow isochronous (X---X---)	400	20, 22.5, 28.4, 40.2	Target in baseline discrimination session
(ii) Fast isochronous (X-X-X-X-)	200	20	Non-target in baseline discrimination session
(iii) Galloping (XXX-XXX-)	100	20, 22.5, 28.4, 40.2	Non-target in baseline discrimination session
(iv) ABA- (ABA-ABA-)	100	A: 20; B: 22.5, 28.4, 40.2 (ΔF = 2, 6, 12 semitones)	Test stimulus in probe session

**Experimental procedure.** The behavioral experiments consisted of 3 steps; an initial shaping session, baseline discrimination session, and probe session [24].

In the initial shaping session, rats first learned the place of food pellets. Rats were then trained to poke the left hole during lighting of the green-colored LED in the hole. In the first 100 trials, poking to the left hole was rewarded with a food pellet. On the following day, a reward food pellet was given when poking to the left hole was followed by poking to the right hole within 5 s. This shaping session was terminated when this set of poking was achieved, and the baseline discrimination session started on the following day.

In the baseline discrimination session, rats were trained to discriminate the slow isochronous sequences from either the fast isochronous sequence or galloping sequences. In this task, rats were trained for 3-4-h per day. The green LED in the left hole was illuminated when a new trial was ready to start. At this moment, only the left hole was accessible to rats and the right hole was inaccessible due to the closure of the sliding door. To initiate a trial, rats had to poke the left hole, which was followed by presentation of a 8-s tone sequence stimulus. The test stimulus was either the slow isochronous, fast isochronous or galloping sequences. During the first 5 s of the test stimulus, rats were not still allowed to poke the right hole. After this 5-s observation period, the door opened and the right hole became accessible for another 5 s. At poking to the right hole during the 5-s access period, the test stimulus stopped and the door at the right hole closed; then, either food reward or a feedback signal of 2-s, 800-Hz tone was delivered. Poking at the presentation of slow isochronous sequence, i.e., the target sequence, triggered delivery of food reward (hit poking). False-positive poking for the non-target sequences, i.e., either the fast isochronous or galloping sequences, was followed by a feedback tone. For the non-target sequences, rats had to wait without poking for 5 s from the door opening; no reward was delivered for this correct behavior. No poking for the target sequence within the 5-s access period was followed by the door closure at the right hole and the delivery of feedback tone. After the door closure at the end of every trial, 8-s time-out period was made between trials. A trial with an identical stimulus repeated until rats performed correctly. After the correct performance, a test sequence in a next trial was chosen pseudorandomly. When the hit poking rate exceeded 65% for 3 days or more, the baseline discrimination session was terminated.

On the following day of termination of the baseline discrimination session, a series of probe sessions were initiated, where behaviors in the presence of ABA- sequences were tested. In the probe session, ABA- sequences were presented in 20% of trials, in which neither reward nor feedback signal was provided; the remaining trials were identical to those in the baseline discrimination session with aforementioned stimulus-response-outcome association. The probe session evaluated how the poking rate depended on ΔF of ABA- sequence (Table 1). A high poking rate was taken as a possible sign that a slow isochronous sequence was segregated in perception of rats. The evaluation in the probe session was considered valid only when the hit poking rate to slow isochronous sequences was 65% or more on a test day; otherwise, data gathered on this day were excluded. In the probe session, rats were tested for 3-4-h per day, during which ∼400 trials were conducted. The probe session lasted for a few weeks, during which at least 200 trials were tested for each ΔF.

### Electrophysiology

**Animal preparation.** Nine adult male Wistar rats at postnatal weeks 11–13 and with a body weight of 330–380 g were used in the experiments. These rats were naïve and had no experience of behavioral experiments. Animals were anesthetized with isoflurane (2.5–3.5% at induction and 1.6–2.0% for maintenance) and held in a fixed position using a custom-made head-holding device. Atropine sulfate (0.1 mg/kg) was administered at the beginning of the surgery and every 7 h thereafter to reduce the viscosity of bronchial secretions. A heating blanket was used to maintain body temperature at around 37°C. The temporal muscle, cranium, and dura overlying the right auditory cortex were surgically removed, and the exposed cortical surface was filled with saline to prevent desiccation. Cerebral edema was minimized by drainage of the cisternal cerebrospinal fluid. The respiratory rate, heart rate, and hind paw withdrawal reflexes were monitored to maintain a stable adequate anesthetic level throughout the recording procedure. In addition, a small craniotomy was performed near the bregma to embed a reference electrode with an electrical contact to the dura mater. The ground electrode was placed under the cervical neck skin. The right eardrum was ruptured and waxed to ensure unilateral sound inputs from the ear contralateral to the exposed cortex.

**Recording and stimulation.** All electrophysiological recordings were performed in a sound-attenuated room (AMC-4015; O’hara & Co. Ltd., Tokyo, Japan). Microelectrode arrays with a grid of 10×10 recording sites and interelectrode distances of 400 µm (ICS-96 Array; Blackrock Microsystems Inc., Salt Lake City, UT) were used, and neural signals were simultaneously obtained from 96 electrodes (4 corner electrodes were excluded from the analysis). Electrode impedances were approximately 120 kΩ under 1-kHz, 0.1-V sinusoidal waves.

LFPs were obtained with an amplification gain of 1000, a digital filter passband of 0.3–500 Hz, and a sampling frequency of 1 kHz (Cerebus data acquisition system; Cyberkinetics Inc., Salt Lake City, UT). The spatial distribution of click-evoked potentials was first mapped on the cortical surface to identify the location of the auditory cortex. The largest focal activation to a click is obtained at the center of anterior auditory cortex [25], serving as a landmark for the appropriate positioning of the electrode array. The primary auditory cortex was identified on the basis of the short post-stimulus latency in the most dorsal auditory field containing a complete high-to-low tonotopic gradient running along the anterior-to-posterior axis [25], [26]. The arrays were inserted to a depth of about 700 µm below the pial surface to measure LFPs.

A function generator (WF1946; NF Corp., Kanagawa, Japan) presented tonal stimuli in a free field through a speaker (EAS-10TH800; Panasonic Corp., Osaka, Japan) positioned 10 cm anterior to the left (contralateral) ear. The frequency and intensity of test stimuli were calibrated at the pinna with a microphone (4939; Brüel & Kjær, Nærum, Denmark) and a spectrum analyzer (CF-5210; Ono Sokki Co., Ltd., Kanagawa, Japan).

Prior to the main experiments, tone bursts with varied frequencies (2.0, 5.0, 10, 20, 32, 40, and 50 kHz) were delivered every 1 s to identify the local activation focus for each test frequency, i.e., best frequency (BF) site. The test intensity was fixed at the 70 dB sound pressure level (SPL; in decibels with respect to 20 µPa), and the duration, plateau, and linear rise/fall times were 30 ms, 20 ms, and 5 ms, respectively.

The main experiments used both the ABA- and A-A- sequences. In the ABA- sequence, 2 tones with different frequencies (tones A and B) were presented alternately (ABA-). The A tones preceding and following the B tones were referred to as A_1_ tones and A_2_ tones (i.e., A_1_BA_2_-), respectively. When the B tone is identical to A tone (i.e., ΔF = 0; AAA-AAA-), the stimulus sequence was supposed to be inevitably perceived as a single stream, and therefore, referred to as the integrated stream, or *Integration*, condition (Fig. 1 **B**). ABA- sequences other than the *Integration* condition, i.e., ΔFs > 0, were referred to as the *ABA-* condition for convenience. The A-A- sequence was made by removing B tones from the ABA- sequence. This sequence could be perceived identically as a segregated stream, and therefore referred to as the segregated A-stream, or *Segregation*, condition.

Both A and B tones were tone bursts with 70 dB SPL, 30-ms duration, and 5-ms linear rise/fall ramps. In accord with the behavioral experiments, the frequency of the A tone was constant at 20 kHz, whereas that of the B tone was manipulated between 20 kHz and 40 kHz in semitone increments (i.e., 6% frequency difference to the reference tone; Table 2). ITI—the time interval between onsets of successive tones in the ABA triplet—varied between 100, 150, and 200 ms. The interval separating successive triplets and that between onsets of successive tones in the A-A- sequence was double the ITI. The duration of each tone sequence was 8 s, and the inter-sequence interval was 4 s. Each tone sequence was presented 20 times in total in a pseudorandom order.

**Table 2:** Test frequencies used in the physiological experiments.

	*ABA*- condition					*Integration* condition	*Segregation* condition
A tone (kHz)	20	20	20	20	20	20	20
B tone (kHz)	22.5	25.2	28.4	31.9	40.2	20	-
ΔF (semitone)	2	4	6	8	12	0	-

**Data analysis.** Neural signals were investigated in detail at a recording site, where the A tone (20-kHz tone) elicited the maximal response in the primary auditory cortex. The 10×10 grid array was able to cover the whole area of tone responsive region, allowing easy identification of this test site. Because auditory streaming is likely to occur after several seconds of the sequence onset [4], [27]–[29], neural signals during presentation of tone sequence were equally divided into 3 time periods, i.e., initial, middle, and final thirds of the neural signals; the final thirds of these signals were mainly analyzed.

First, for the A_1_ and A_2_ tones, the peak amplitudes of evoked LFPs were quantified as a function of ITI and ΔF. Perceptual streaming was inferred with similarity index (SI) that evaluated whether and how each condition was close to either *Integration* or *Segregation* conditions. SI was defined as follows:
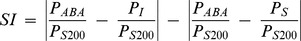
(1)where *P_ABA_*, *P_I_* and *P_S_* denoted the peak amplitudes under *ABA-*, *Integration* and *Segregation* condition, respectively, at a given ITI. *P_S_*_200_ denoted the peak amplitude of *Segregation* condition with 200-ms ITI, which was supposed to be the largest evoked response and thus served as a reference value to normalize *P_ABA_*, *P_I_* and *P_S_*. This normalization allowed comparison across subjects. When *P_ABA_* was identical to *P_S_*, SI yields the maximum, *P_S_*/*P_S_*_200_ – *P_I_*/*P_S_*_200_ (> 0; *P_S_* > *P_I_*); on the other hand, when *P_ABA_* was identical to *P_I_*, SI yields the minimum, i.e., *P_I_/P_S_*_200_ – *P_S_*/*P_S_*_200_ (< 0). Thus, positive SI suggests that the given condition is close to *Segregation* condition in terms of peak amplitude, while negative SI suggests *Integration* condition. The distributions of SI in the ITI–ΔF plane examined whether the neural signal under test was a neuronal correlate of van Noorden’s perceptual boundary.

**Figure 2:**
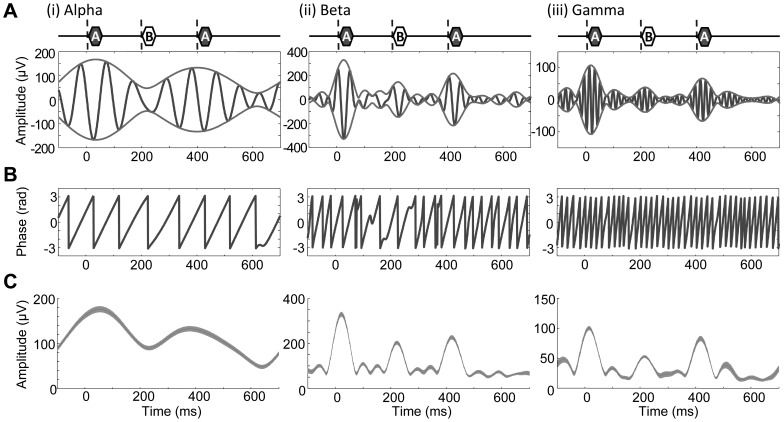
Characterization of the amplitude and phase of LFP. **A.** Filtered signals and their envelopes in response to a representative trial of an ABA triplet: (i), alpha band; (ii), beta band; (iii), gamma band. The timings of A_1_, B and A_2_ tones are indicated above each inset. **B.** Phases. **C.** Amplitude envelopes. The mean and standard deviation (s.d.) across trials are given by a shaded band.

Second, the measured LFP was split into 3 frequency bands by digital bandpass filtering: alpha, 8–13 Hz; beta, 13–30 Hz; and gamma, 45–60 Hz. To determine these bands, spectrograms of LFP were obtained during the presentation of ABA- sequences and these spectrograms were averaged across subjects. This time-frequency analysis employs the following parameters: sampling frequency, 400 Hz; FFT window size, 128 points; step size of the sliding window, 32 points. The digital filters used were linear-phase, finite-impulse response (FIR) filters, which were designed using the Parks-McClellan algorithm (Matlab; Mathworks Inc., Natick, MA) such that the attenuation at 90% of the low band edge and 110% of the high band edge was 40 dB. Filtered signals were converted into analytic signals of instantaneous amplitude and phase using the Hilbert transform (Fig. 2). For each frequency band, the peak amplitude was the grand average of analytic amplitude, from which SI was calculated according to equation (1). Phase locking was quantified by inter-trial phase coherence (ITPC) on the basis of the phase distribution across trials for every combination of ITI and ΔF under test:
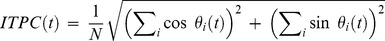
(2)


where *N* is the number of trials and *θ_i_*(*t*) is the instantaneous phase at time *t* of *i* ^th^ trial. ITPC is the subtraction of circular variance from 1 [30], [31]. ITPC ranged between 0 and 1. If the phase at the specific time varies a little across trials, ITPC is close to 1; otherwise, it is close to 0. ITPC values were characterized within 1–20 ms post stimulus latency, and the average during this test period was used to evaluate phase modulation by each tone. Similar to the peak amplitude, SI of ITPC was obtained as follows:
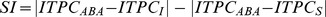
(3)


where *ITPC_ABA_*, *ITPC_I_*, and *ITPC_S_* represent ITPC under *ABA-*, *Integration*, and *Segregation* condition, respectively.

In the van Noorden’s perceptual boundary, segregated stream perception is more likely to be induced by a short ITI. To examine whether neural characteristics related to such a property was observed in the SI distribution, the difference between SI at 100 ms ITI and SI at 200 ms ITI was defined as ΔSI at a specific ΔF condition. To evaluate whether these SI characteristics contributed to the build-up of auditory streaming [4], temporal evolution of the SI distribution in ITI–ΔF plane was investigated by comparing the SI distributions in the initial, middle, and final thirds of tone sequences (0–2.4 s, 2.4–5.2 s, and 5.2–8.0 s from sequence onset, respectively).

## Results

### Behavioral experiment

**Figure 3:**
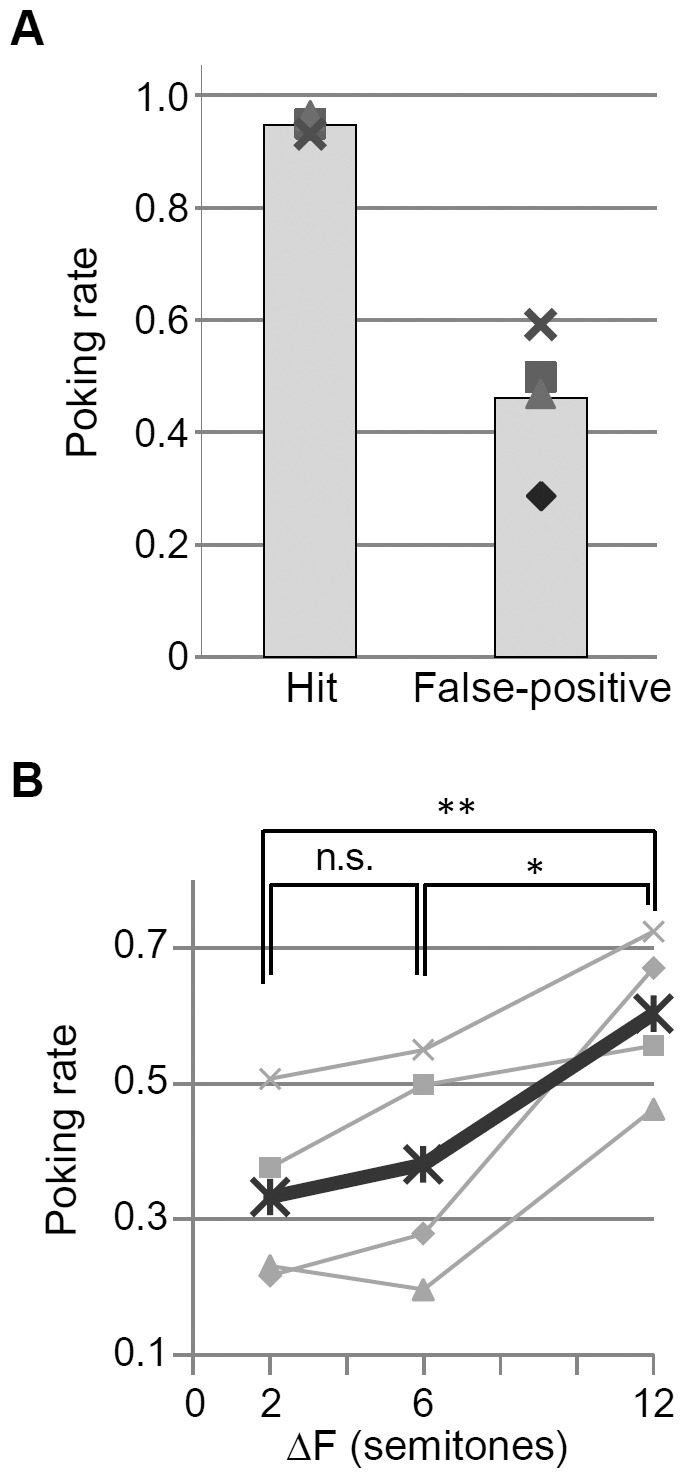
Behavioral evaluation of auditory streaming. **A.** Baseline discrimination session. Poking was rewarded in the presence of slow isochronous sequence, but not in the fast isochronous and galloping sequences (Table 1). Hit and false-positive poking rates are depicted for individual subjects (symbols) and the average (bars). **B.** Probe session. Poking rates are depicted with respect to ΔF of ABA- sequence for individual subjects (thin lines) and the average (bold line).

Figure 3**A** shows the hit and false-positive rates of poking over the last three days in the baseline discrimination session, where rats were trained to discriminate the slow isochronous sequence. The hit poking rate was almost 95% on average across all the subjects and ΔFs, whereas the false positive rate reduced to almost the same level as the chance. In closer investigation, the false positive rates to galloping sequences and fast isochronous sequences were 0.31±0.15 and 0.56 ± 0.078 (mean ± s.d.), respectively, indicating that rats were able to behave correctly to galloping sequences but were likely to confuse fast isochronous sequences with the target of slow isochronous sequences. These results suggest that rats were successfully trained so as to discriminate the slow isochronous sequences from galloping sequences.

Figure 3**B** shows poking rates in the probe session as a function of ΔF in ABA- sequences, indicating that the poking rate monotonically increased with ΔF. Statistically, the poking rate at ΔF = 12 was significantly larger than those at ΔF = 2 and 6 (one-way ANOVA, *F*(2,9)  = 4.38, *p* = 0.0470; *post hoc* one-sided *t*-test after Bonferroni correction, *p* = 4.0E-3 and 0.0366). These results could be taken as a proof that rats extracted the slow isochronous sequence from ABA- sequence with short ITI and large ΔF, corroborating the ΔF-dependent stream segregation in the human psychophysics [4], [32]. Thus, rats could be placed as an adequate animal model to investigate neural mechanisms of stream segregation in a given range of test parameters of frequency and ITI.

### Tone-evoked LFP

**Figure 4:**
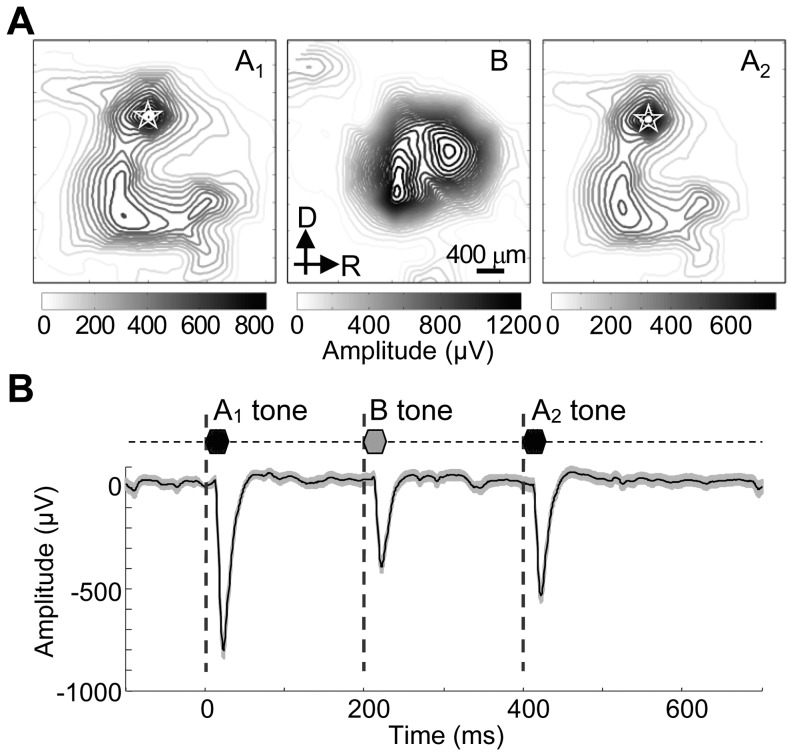
Representative LFP evoked by ABA triplet. **A.** Cortical mapping of evoked LFP maxima to A_1_, B and A_2_ tones. The test ΔF was 12 semitones, and ITI was 200 ms. A white star indicates the local focus of A tone-evoked response in the primary auditory cortex. **B.** Evoked LFPs to a triplet of an ABA- sequence at the local focus of A tone-evoked response. A representative grand average across trials and s.d. are given.

In total, 407 recording sites were tone responsive in the 9 cortices tested. Figure 4**A** shows a representative spatial distribution of LFP peak amplitudes to ABA triplets with ΔF of 12 semitones and ITI of 200 ms. A and B tones elicited different spatial distributions, where the B tone-evoked response was obtained at the center of auditory cortex (Fig. 4**A**, middle panel), while the A tone-evoked response was observed in locations surrounding the B tone-responsive sites (Fig. 4**A**, left and right panel) according to the tonotopic gradient [26], [33]. In the cortical map of each subject, a recording site with BF matching the A-tone frequency (20 kHz) was reliably identified within the primary auditory cortex. Thus, 9 recording sites, i.e., one site from each subject, were used. Figure 4**B** shows a representative grand average of tone-evoked LFP to an ABA triplet with ΔF of 6 semitones and ITI of 200 ms. The negative polarity of LFP indicates that the microelectrode array was located at cortical layer 4 or deeper. The B tone-evoked LFP was smaller than the A tone-evoked LFP because the B tone frequency was not BF for the test site. The A_2_ tone-evoked LFP was smaller than the A_1_ tone-evoked LFP because of forward suppression by the B tone.

**Figure 5:**
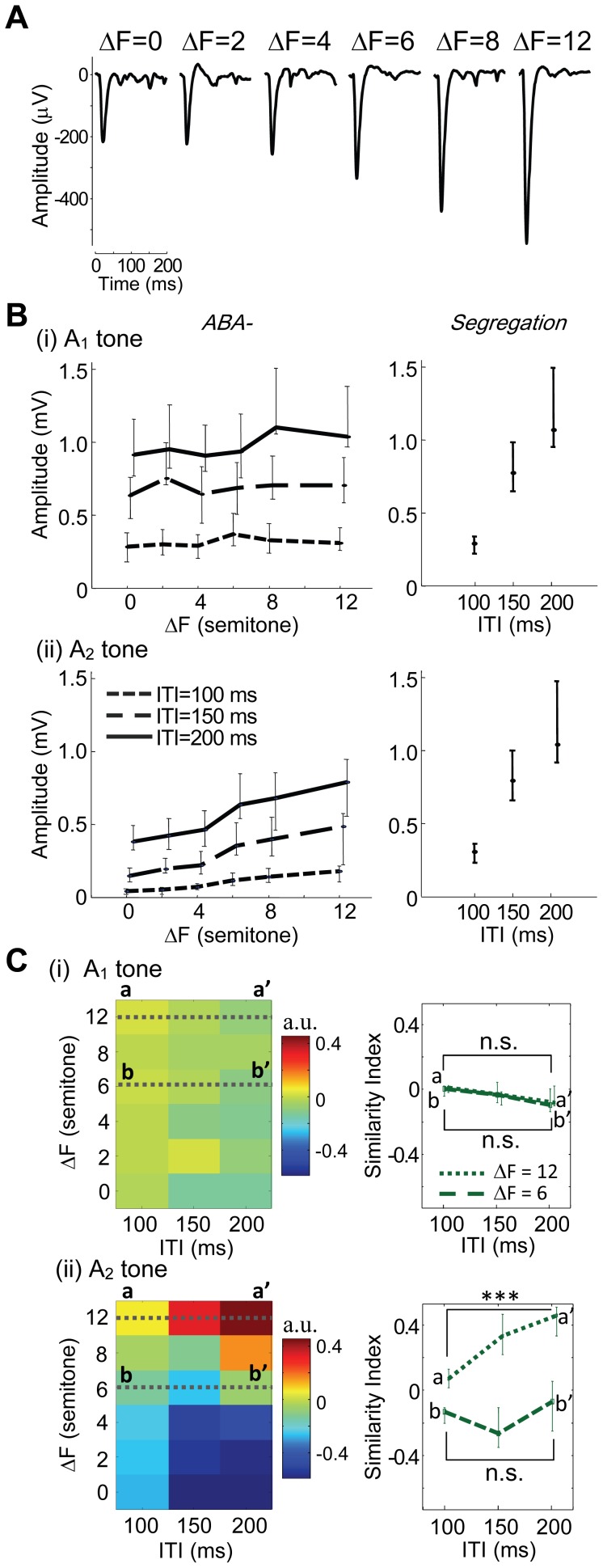
Characterization of the peak amplitudes of an unfiltered LFP. **A.** ΔF-dependent A_2_ tone-evoked LFPs at BF site for the A tone. The test ITI was 200 ms. **B.** Peak amplitudes as a function of ΔF and ITI for A_1_ tone-evoked response (i) and A_2_ tone-evoked response (ii). Left and right columns correspond to *ABA*- condition and *Segregation* condition, respectively. The type of lines indicates ITI: dotted, 100 ms; broken, 150 ms; solid, 200 ms. The medians and quartile deviations are given. **C.** SI of peak amplitude. Left column depicts SI distribution in the ITI–ΔF plane. Right column depicts SI at specific ΔFs: a-a’, 12 semitones; b-b’, 6 semitones. Asterisks indicate statistical significance of SI between ITI of 100 and 200 ms (Two-sided *t*-test): *, *p*<0.05; **, *p*<0.01; ***, *p*<0.001.

Figure 5**A** shows a representative average of A_2_ tone-evoked LFPs at the test site as a function of ΔF. The response amplitude was minimum when the B tone was identical to the A tone, i.e., ΔF = 0, and monotonically increased with ΔF. Figure 5**B** quantifies the LFP peak amplitude in response to the A_1_ and A_2_ tones with respect to all ΔF and ITI conditions tested. The A_1_ tone-evoked LFP increased monotonically with ITI but was independent of ΔF (Fig. 5**B**, (i), left inset). The *Integration* (i.e., ΔF = 0) and *Segregation* conditions (i.e., A-A- sequence) also elicited comparable responses (Fig. 5**B**, (i), right inset). When ITI was identical, the A_1_ amplitude exhibited no statistical difference according to the B tone in all the conditions of *ABA-* and *Integration* (one-way ANOVA: 100 ms ITI, *F*(5,48)  = 0.257, *p* = 0.934; 150 ms ITI, *F*(5,48)  = 0.0614, *p* = 0.997; 200 ms ITI, *F*(5,48)  = 0.0794, *p* = 0.995). Thus, the A_1_ tone-evoked LFP was not the neural correlate of auditory stream segregation. In contrast, ΔF and ITI were likely to serve as substantial parameters to influence the A_2_ tone-evoked LFPs (Fig. 5**B**, (ii)). First, the A_2_ tone-evoked LFPs increased with ITI just as the A_1_ tone-evoked LFPs. Second, and more importantly, the A_2_ tone-evoked LFPs monotonically increased with ΔF (Fig. 5**B**, (ii), left inset), thereby approaching the *Segregation* condition (Fig. 5**B**, (ii), right inset) and in turn being apart from the *Integration* condition. Statistically, the A_2_ tone-evoked LFPs depended on ΔF (one-way ANOVA: 100 ms ITI, *F*(5,48)  = 13.24, *p*  = 4.06×10^−8^; 150 ms ITI, *F*(5,48)  = 9.23, *p* = 3.30×10^−6^; 200 ms ITI, *F*(5,48)  = 6.20, *p*  = 1.64×10^−4^).

These results are generally consistent with the psychophysical finding that large ΔFs are more likely to be perceived as segregated streams, possibly due in part to forward masking. First, temporal masking becomes less effective with increasing ITI, i.e., the time interval between masker and maskee, resulting in increased A_1_- and A_2_- evoked responses. Second, the masking effectiveness also depended on the frequency of masker: the A_1_ -evoked response was predominantly masked by A_2_ of the preceding triplet, and was therefore independent of ΔF because the frequency of both A_1_ and A_2_ tones was maintained constant among conditions. In contrast, the A_2_ tone-evoked LFP was affected by the B tone immediately preceding it, i.e., it was significantly dependent on ΔF.

In Fig. 5**C**, (i) and (ii) show SI of the A_1_- and A_2_-evoked LFPs for every condition of ITI and ΔF, respectively. SI to the A_1_ tone was independent of ITI and ΔF, further indicating that the A_1_ tone-evoked responses were not neural correlates of auditory stream segregation. In contrast, SI to the A_2_ tone had a distinct distribution that gradually changed from an *Integration* condition-like state to a *Segregation* condition-like state with increased ΔF, corroborating van Noorden’s psychophysical findings. This trend in the A_2_ tone-evoked LFP was enhanced with increased ITI (Fig. 5**C**, (ii), right inset); in the A_2_ tone-evoked LFPs, SI under ITI of 200 ms was significantly larger than that under an ITI of 100 ms at a ΔF of 12 semitones (one-way ANOVA, *F*(2,24)  = 7.21, *p* = 0.004; *post hoc* two-sided *t*-test, *p* = 1.0×10^−4^). These response properties were substantially inconsistent with van Noorden’s perceptual boundary (Fig. 1 **B**), i.e., a segregated stream would be more likely with the combination of larger ΔFs and shorter ITIs. Thus, auditory stream segregation was not entirely correlated with the A_2_ tone-evoked LFP.

### Band-specific LFP

**Figure 6:**
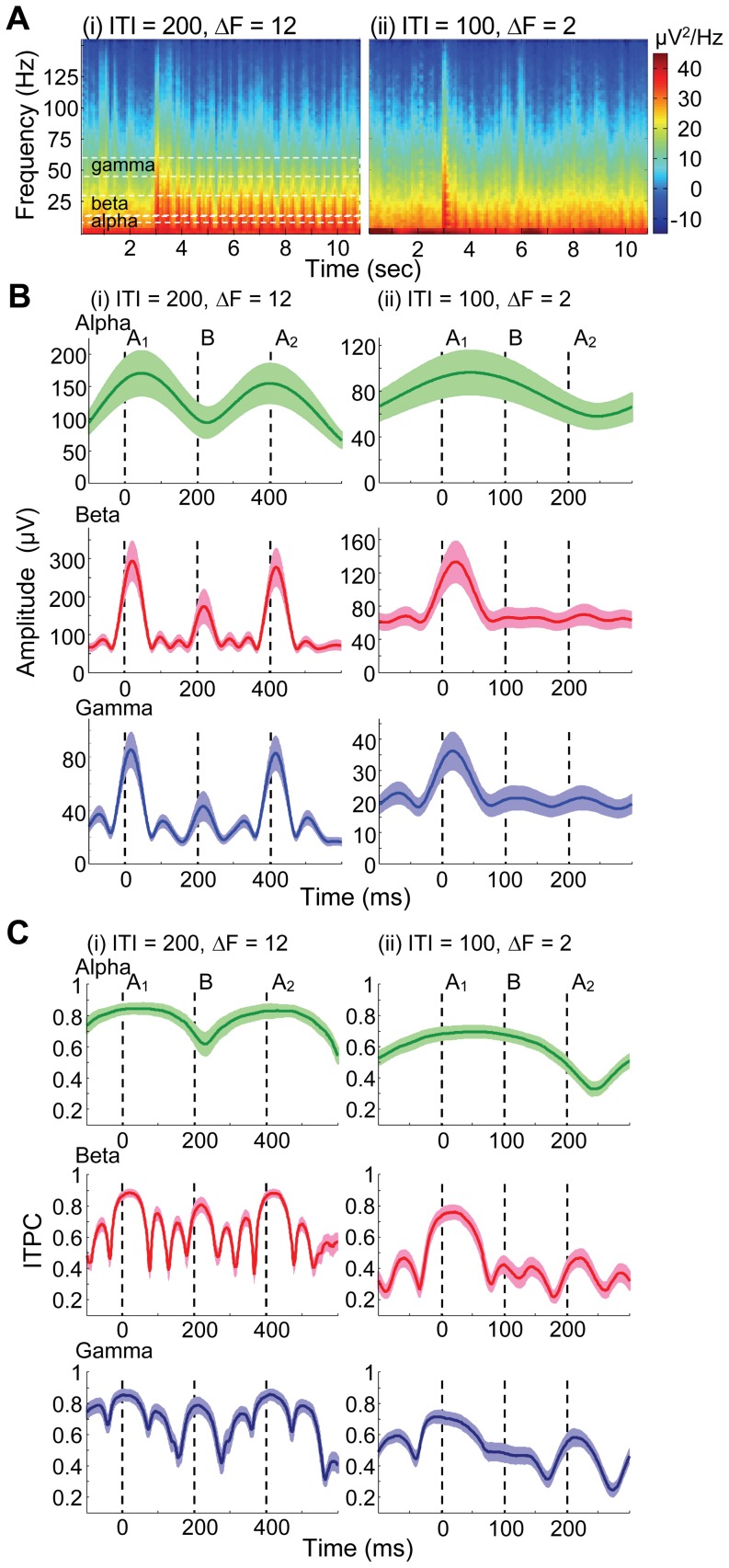
Band-specific LFP to be tested. **A.** Spectrograms during the presentation of ABA- sequence: (i), ITI = 200, ΔF = 12; (ii), ITI = 100, ΔF = 2. The averages across subjects are shown. The onset of test sequences was 3 s. Broken lines indicate the boundary of test frequency bands of alpha, beta and gamma. **B.** Amplitudes in response to ABA triplet. Alpha, beta and gamma bands are shown. The mean across subjects and s.d. are given. The broken lines depict stimulus onsets. **C.** ITPCs in response to ABA triplet.

Figures 6**A** show LFP spectrograms averaged across all the subjects during presentation of ABA- sequence under representative conditions. When ITI was long (200ms) and ΔF was large (12 semitones), both repetitive A_1_ and A_2_ tones elicited stimulus-locked LFPs with a broad band ranging from theta (< 6 Hz) to high-gamma (> 60) bands (Fig. 6**A**, (i)). On the other hand, when ITI was short (100 ms) and ΔF was small (2 semitones), A_2_ tones tended to elicit much less stimulus-locked responses than A_1_ tones (Fig. 6**A**, (ii)). Furthermore, the stimulus locking was likely dependent on conditions in a band-specific manner; for example, the stimulus locking tended to decrease with frequency more rapidly in Fig. 6**A**, (ii) than in Fig. 6**A**, (i).

Figures 6**B and 6C** show analytic amplitudes and phases, respectively, in band-specific LFPs averaged across subjects in response to ABA triplets during the latter half of the given sequences. In all bands tested, a small ΔF and short ITI decayed the band-specific amplitudes to both B and A_2_ tones, which were consistent with those in unfiltered LFP due to forward masking. The phases in the beta and gamma bands were consistently locked (ITPC > 0.8) to either A_1_ or A_2_ tone typically within a post stimulus latency of 20 ms, although some fluctuations of ITPC were observed in the following inter-tone period; ITPC was thus characterized as the average during 1 – 20 ms in our analyses. The phase locking to A_2_ tone was higher in the long ITI, large ΔF conditions than in the short ITI, small ΔF conditions. In the alpha band, the phase locking to A_2_ was observed in the long ITI (200 ms) condition, but not in the short ITI (100 ms) condition, because the cycle of alpha band was too long to be flexibly timed to an upcoming stimulus onset.

**Figure 7:**
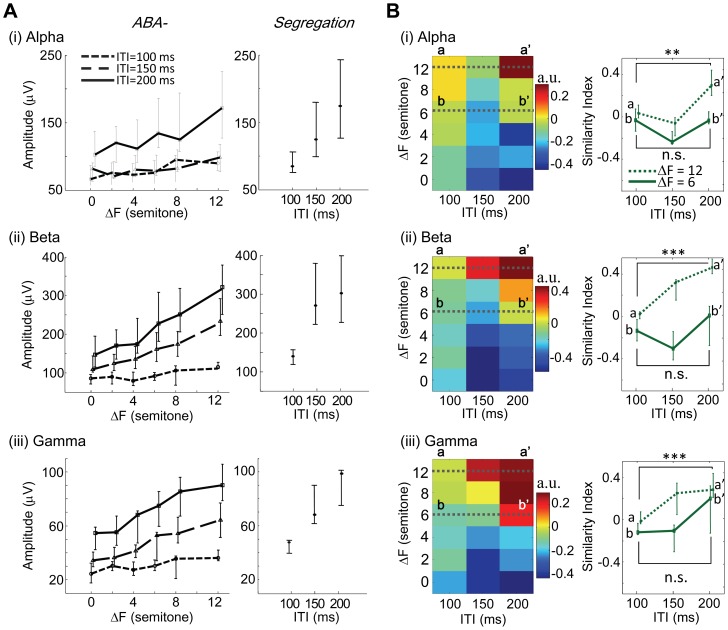
Characterization of band-specific LFP amplitude to A_2_ tone. **A.** Peak amplitudes as a function of ΔF and ITI for alpha (i), beta (ii), and gamma bands (iii). **B.** SI of peak amplitude. Conventions comply with Fig. 5.

**Amplitude property in ITI-ΔF plane.** Figures 7**A** shows that, in all the bands tested, the band-specific LFPs to A_2_ were dependent upon ITI and ΔF in a similar manner to the unfiltered A_2_ tone-evoked LFPs (Fig. 5). For each ITI, the peak amplitude approached the *Segregation* condition with an increased ΔF, and in turn dissociated from the *Integration* condition. Consequently, the SI distributions in an ITI–ΔF plane exhibited common characteristics irrespective of frequency bands (Fig. 7**B**), which were similar to the SI distribution of the unfiltered A_2_ tone-evoked LFP (Figs. 5**C**, (ii)): as ΔF increased, the responses gradually changed from *Integration* condition-like to *Segregation* condition-like, although the most pronounced change was associated with the longest ITI. As was seen with the unfiltered LFP, in all the bands tested, SI of the band-specific LFPs to the A_2_ tone were significantly larger with long ITI (200 ms) than short ITI (100 ms) (one-way ANOVA was applied across all ITI conditions (100, 150, 200 ms): alpha, *F*(2,24)  = 17.8, *p*<10^−4^; beta, *F*(2,24)  = 14.0, *p*<10^−4^; gamma, *F*(2,24)  =  7.91, *p* = 0.002. *Post hoc* two-sided *t*-test: alpha, p = 0.0013; beta, *p*<10^−6^; gamma, *p*<10^−4^), indicating that the frequency band amplitude was not the neural correlate of auditory stream segregation.

**Figure 8:**
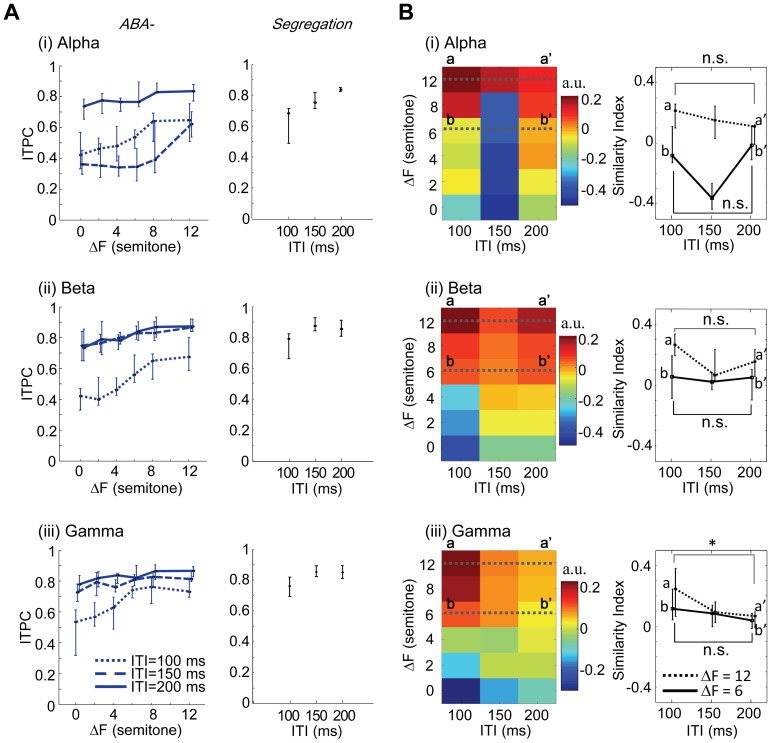
Characterization of band-specific ITPC to A_2_ tone. **A.** ITPCs as a function of ΔF and ITI for alpha (i), beta (ii), and gamma bands (iii). **B.** SI of ITPC. Conventions comply with Fig. 5.

**Stimulus phase-locking properties in ITI-ΔF plane.** Phase coherence of evoked responses was investigated in Fig. 8 as a possible candidate of the neural correlates of auditory streaming. Similar to the response amplitude, in all the test frequency bands, ITPC to A_1_ monotonically increased with ITI but was not influenced by ΔF (data not shown), and therefore, was not correlated with auditory streaming. On the other hand, ITPC to A_2_ depended nonlinearly on ITI and ΔF in a band-specific manner (Fig. 8**A**). As was seen with response amplitude (Figs. 5 and 7), ITPC of the ABA- sequence in all the test bands increased with ΔF and became *Segregation* condition-like. Whereas ITPC in all the bands was largest at the long ITI condition across ΔFs, particularly in the beta and gamma bands, the ΔF-dependent change in ITPC was most pronounced at short ITI, which was substantially different from the trends associated with either unfiltered or band-specific amplitude (Figs. 5 and 7). The remarkable changes in ITPC at short ITI were not associated with the alpha band, where ITPC changed non-monotonically with ITI.

Consequently, in the beta and gamma bands we found that the SI distributions of ITPC to A_2_ in ABA- sequence changed from *Integration* condition- to *Segregation* condition-like phase locking as ΔF increased, especially with short ITI (Figs. 8**B**, (ii) and (iii)). In fact, statistical analyses proved that SI of ITPC in the gamma-oscillation to A_2_ was significantly greater at 100 ms ITI than 200 ms ITI, at ΔF of 12 semitones (Figs. 8**B**, (iii), right inset; one-way ANOVA, *F*(2,24)  = 5.69, *p*<0.01; *post hoc* two-sided *t*-test, *p* = 1.14 × 10^−2^), while the SI did not significantly increase with ITI at ΔF of 6 semitones (one-way ANOVA, *F*(2,24)  = 2.75, *p* = 0.084; *post hoc* two-sided *t*-test, *p* = 0.074). SI of the beta-band at ΔF of 12 semitones also tended to be larger in short ITI than that in long ITI, although the tendency was not significant (Figs. 8**B**, (ii), right inset; one-way ANOVA, *F*(2,24)  = 2.02, *p* = 0.155; *post hoc* two-sided *t*-test, *p* = 0.159) and slightly non-monotonic (SI at 150 ms ITI < SI at 200 ms ITI); at ΔF of 6 semitones, SI did not depend on ITI (one-way ANOVA, *F*(2,24)  = 0.250, *p* = 0.781; *post hoc* two-sided *t*-test, *p* = 0.587). With the long ITI, SI in the gamma band was similar for all ΔFs, corresponding to the psychophysical findings of poor ΔF-dependency in van Noorden’s perceptual boundary (Fig. 1**B**) as well as an ambiguous perceptual state at long ITI. Thus, the behavior of SI in the higher frequencies, particularly the gamma band, closely resembled van Noorden’s perceptual boundary (Fig. 1**B**).

In contrast, SI in the alpha band was non-monotonically dependent upon ITI with a local minimum at 150 ms, which was not consistent with the psychophysical findings (e.g., at ΔF of 6 semitones, one-way ANOVA: *F*(2,24)  = 7.00, *p* = 4.00 × 10^−3^). There was no significant difference in SI in the alpha band between 100 ms and 200 ms ITIs, at ΔF of 12 semitones (Fig. 8**B**, (i), right inset; one-way ANOVA, *F*(2,24)  = 0.472, *p* = 0.630; *post hoc* two-sided *t*-test, p = 0.205). This is consistent with phase locking to stimulus onset in the higher frequency bands being a neuronal correlate of auditory stream segregation.

**Figure 9:**
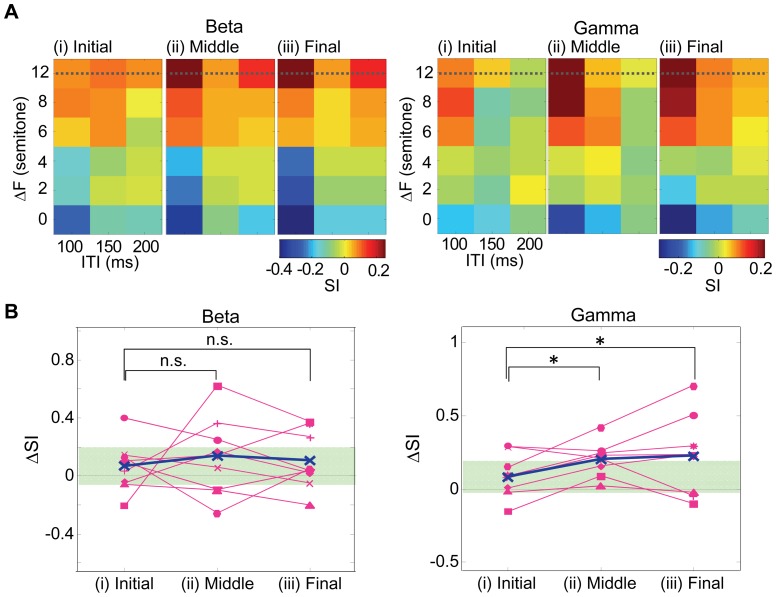
Temporal evolution of beta- and gamma-band ITPC to A_2_ tone. **A.** SI distributions in the ITI–ΔF plane: left panel, beta band; right panel, gamma band. SIs were quantified in the initial (i), middle (ii), and final thirds (iii) of the tone sequence. **B.** ΔSI (i.e., an increment from SI at 200 ms ITI to SI at 100 ms ITI) at 12 semitones of ΔF (dotted line in **A**) in the initial, middle and final thirds of tone sequences. Data from individual animals are shown. Bold line denotes the average ΔSI across subjects. Asterisks indicate statistical significance of ΔSI between the initial period and the other periods at 12 semitones of ΔF (*p*<0.05). Shaded areas correspond to 95% confidence interval of ΔSI distribution during the initial period.

Based on our hypothesis, neural correlates should evolve temporally according to the build-up effect. To test this in our system, we quantified SIs of the beta- and gamma-bands ITPC in the initial (∼2.4 s), middle (∼5.2 s), and final thirds (∼8.0 s) of the tone sequence (Fig. 9**A**). The duration of the final third is analogous to the duration required for the build-up of auditory streaming in human psychophysical studies [4], [34]. We found that SIs of ITPC in the middle and final thirds of the tone sequence were more *Segregation* condition-like at short ITI and large ΔF than in the initial third, particularly in the gamma band. For ΔF of 12 semitones, Fig. 9**B** quantifies an increment from SI at 200 ms ITI to SI at 100 ms ITI as ΔSI in a given period of test tone sequences. In the gamma band, the ΔSI in the middle and final third were significantly larger than the ΔSI in the initial third (one-sided paired *t*-test with Bonferroni–Holm correction for multiple comparisons, *p*<0.05), whereas there was no significant change associated with the beta band (all tested combinations had corrected *p* values > 0.36). This suggests that the gamma-band SI showed the build-up-like behavior after around 2.4 s from sequence onset.

These data show that the phase locking to stimulus onset in the gamma-band oscillation was consistent with the requirements for a neuronal correlate of auditory streaming in terms of the perceptual boundary and build-up properties.

## Discussion

### Summary of findings

Our aim was to investigate the relationship between the stimulus-locked phase modulation of cortical oscillation and the perceptual formation of an auditory stream. Our behavioral evaluation provided compelling evidences for the first time that rats are able to organize auditory stream. We then investigated LFPs on cortical layer IV or deeper in the primary auditory cortex of anesthetized rats to examine temporal locking at a local neuronal population level, rather than the level of unit activities. We focused on LFPs to A tones in the ABA- sequence at a BF site of A-tone frequency. We found that with large ΔFs and short ITIs the LFP phase locking to stimulus in higher frequency bands, particularly in the gamma band, was able to better describe van Noorden’s perceptual boundary than the LFP amplitude. Furthermore, the tendency was significantly observed after around 2.4 s from sequence onset. These results support our hypothesis that temporal modulation of cortical oscillations should be considered as neurophysiological mechanisms of auditory streaming, in addition to forward suppression, tonotopic separation, and multi-second adaptation.

### Methodological consideration

**Behavioral experiments.** Our behavioral test could be taken as an evidence of auditory streaming in rats; however, relatively high false-positive rates (> 0.4) in the baseline discrimination session hampered precise estimation of streaming effects under each condition, specifically at small ΔF, where test sequences were acoustically close to non-target, galloping sequences (Fig. 3). This high false-positive rate was possibly due to our simple design of go/no-go task, where the correct no-go behaviors were not reinforced. To better estimate the perception of streams, target-discrimination task may be preferable; yet, to our experience, it was less successful than the go/no-go task.

This limitation of behavioral test might cause discrepancies between behavioral and physiological results in that the behavioral index exhibited a steep change between 6 and 12 semitones (Fig. 3**B**), while the physiological index showed gradual changes between 0 and 12 semitones (Fig. 8**B** (iii)).

**Contribution of primary auditory cortex.** Recent studies in humans have placed less emphasis on the role of the primary auditory cortex in stream segregation. For example, electroencephalography (EEG) and magnetoencephalography (MEG) studies suggest predominant roles for non-primary auditory cortices [35]–[39]. In addition, perception-dependent activations of fMRI signals during passive listening were more frequently found in the non-primary auditory and association cortices than in the primary auditory cortex [40]–[43]. Nevertheless, thalamo-cortical interactions are involved in the spontaneous switching of perception, suggesting a significant role for the primary auditory cortex [34]. Furthermore, the primary auditory cortex is activated by forced attentive listening to a particular stream, suggesting that volitional control modulates encoding of thalamo-cortical inputs in the primary auditory cortex and substantially affects perception [38], [44]–[47]. These studies provide evidence that encoding of thalamo-cortical inputs in the primary auditory cortex is the substantial underpinning of auditory streaming, and thus its intrinsic properties should be investigated.

**Anesthetic effects.** General anesthesia in our experiments eliminated the possibility of top-down modulatory effects and shed light on the intrinsic thalamo-cortical encoding in the primary auditory cortex. Isoflurane anesthesia potentiates GABA_A_-mediated inhibition and blocks AMPA- and NMDA-induced excitation [48]. In addition, multi-unit activities show a poor temporal following capacity in a repetitive stimulus sequence [49]. Accordingly, with less supra-threshold cortical activity, cortico-cortical feedback inputs are less effective and the thalamo-cortical feed-forward inputs become predominant. Thus, in our study, stimulus-evoked population discharges might not have been accompanied reliably, yet stimulus-induced sub-threshold oscillation could have contributed to establishing a functional basis of stream segregation [50], [51].

The effect of isoflurane should also be band-specific and spatially localized within the cortex. Some studies suggest that the power of the beta (13–30 Hz) and low gamma (30–50 Hz) bands are only slightly affected by isoflurane [52], [53]. Moreover, local gamma coherence remained unchanged whereas long-range, cortico-cortical coherence was attenuated. These data suggest that whereas the influence of isoflurane depended on oscillatory band and spatial localization in cortex, the modulation of low gamma-band oscillation in the local population might be similar under anesthesia and while awake.

**Neurometric index.** In an alternating sequence of ABAB, previous studies derived the neurometric index for streaming from the amplitude ratio of B-tone evoked responses with respect to A-tone evoked responses at a BF site of A-tone frequency [2], [3], [28], [54]. In the ABA- sequence, however, this index became less straight forward because the forward suppression of B tone was only effective to A_2_-tone evoked responses and thereby the amplitude ratio had to be defined with respect to both of A_1_ and A_2_ tones. Thus, instead of characterizing the amplitude ratio, our analysis had focused on A_1_- and A_2_-tone evoked responses. The index for streaming in the present study (i.e., similarity index or SI) was designed under hypothesis that, when A-A- stream was organized from ABA- sequence, neural features under test should approach those in *Segregation* condition and get apart from those in *Integration* condition.

### Possible contribution of LFP amplitude to the streaming effect

We found that the response to A_1_ was unaffected by the B tone because A_2_ followed by an absence made the B tone a less effective masker (Fig. 5**B**, (i)). In contrast, LFPs evoked by A_2_ were dependent on ITI and ΔF (Fig. 5**B**, (ii)), consistent with previous single unit studies [5], [55], [56]. These trends were observed in all band-specific amplitudes of LFP (Fig. 7**B**). Thus, these amplitude behaviors could be accounted for by forward masking. Based on the forward masking, however, the neurometric functions with amplitudes contradict the psychometric function (Fig. 1) in terms of ITI, and exclude their potential as neural correlates of streaming.

### Functional implications of inter-trial phase coherence in the streaming of ABA- sequence

**Phase reliability to stimulus and its contribution to perceptual formation.** ITPC evaluates trial-to-trial temporal coherence in terms of stimulus phase locking, which reflects the reliability and reproducibility of neuronal phase representation for time-varying sensory information. In our study, the smallest ITPC occurred with small ΔF and short ITI, indicating unreliable cortical sensory representation under those conditions and suggesting that phase fluctuation cannot robustly transfer bottom-up input information from thalamus to cortex [57]. Conversely, the combination of large ΔF and short ITI enhanced stimulus phase locking and was comparable to that observed with *Segregation* conditions, possibly indicating that input information was robustly transferred to the cortex and was reliably represented in the cortical oscillation. Furthermore, this kind of phase modulation should be related to perceptual formation, because many studies show that neuronal temporal reliability is closely linked to object representation and simultaneous streaming in addition to response amplitude [18], [44], [58]–[62]. Thus, stimulus-dependent phase modulation could be an appropriate index to investigate the presence of auditory stream formation.

**Differences of neural characteristics between A_1_ and A_2_ tone.** The present findings showed significant ITI- and ΔF-dependent phase modulation to the A_2_ tone, but not A_1_, mainly due to differences in pre- and post-time–frequency properties between A_1_ and A_2_, as discussed previously. The differences in the dependence of A_1_ and A_2_ upon ITI and ΔF should be sensitive to variations of the ABA triplet, possibly contributing to the sequential streaming. These results demonstrated that under conditions favoring *Integration* condition-like perception, the neuronal representation of the ABA triplet was characterized by stronger phase locking to A_1_ and poorer phase locking to subsequent tones in that triplet (Fig. 6**C**). Due to this characteristic difference, the ABA triplet might be perceived as an integrated stream. Conversely, under conditions favoring a *Segregation* condition-like perception, phase locking to A_1_ and A_2_ was similar. If an A-A- sequence was more readily perceived as isochronous when the phase locking to A_1_ and A_2_ was nearly equal, the phase locking under the condition might suggest the formation of isochronous A-tone sequence. Thus, the degree of similarity in phase locking of A_1_ and A_2_ might influence the neural representation of streaming.

**Relationship between oscillation frequency and inter-tone interval.** The alpha band phase coherence changed non-monotonically with ITI, which differed from the behavior of van Noorden’s perceptual boundary. This may be due to perturbation of phase reset, because the frequency range of the alpha band (8–13 Hz) is close, but not equal to the presentation rate at ITI of 150 ms (6.67 Hz). In contrast, the gamma band oscillation was much higher than the highest presentation rate tested in this study (10 Hz), and thus the gamma phase was monotonically dependent on ITI to a greater degree than other frequency bands. In contrast, the beta band was slightly non-monotonic because its oscillation frequency was between the alpha and gamma bands (Fig. 8**B**, (ii), right inset). Therefore, a sufficiently high oscillation frequency with respect to presentation rate of the stimulus might be important for monotonic dependence of the phase modulation on ITI. Considering that stimulus presentation rate would be in the order of tens of milliseconds, even gamma phase locking should be eliminated. However, it should be noted that such short time scales do not favor the psychophysical formation of auditory streaming [63]–[65].

**Functional role in the gamma oscillation phase for streaming.** Perceptual objects are considered to be organized by coherent spatial activity patterns possibly mediated via gamma-band oscillation. Gamma band oscillation is selective to a specific neuronal input [66]. In the present study, the neurometric function with gamma phase coherence might reflect selective phase modulation to A_2_-derived thalamo-cortical input. Furthermore, gamma oscillation is closely related to spiking activities [67], and might contribute to synchrony of spiking activity [68]. Spike synchrony is associated with sensory representation on the basis of the coordinated ensemble activities, which are referred to as a “temporal binding process” in the visual and auditory domains [10], [68]–[73].

Auditory streaming is also considered as one of binding processes because a particular repetitive sequence of tones is temporally bound as an auditory object. In this streaming, neural activities are entrained to the cycle of repetitive components, resulting in stimulus phase locking every cycle. As each stimulus onset was identical across trials, ITPC can be used to evaluate the reliability of phase locking for each cycle in a test sequence. Therefore, in addition to the traditional view that a spatial pattern of gamma oscillation is crucial to the binding process, our results emphasize the importance of temporal reliability of gamma phase locking.

**Evaluation of the phase resetting process with inter-trial phase coherence.** We have shown that ITPCs were nearly equal but the amplitudes were significantly different across conditions, i.e., short ITI/large ΔF vs. long ITI. Using ITPC and an analytic envelope, we were able to evaluate approximately whether a stimulus evoked response was derived from a phase reset or by an additive evoked component. In principle, phase-reset is accompanied by high ITPC and small envelope enhancement, whereas additive evoked components are accompanied by both high ITPC and large envelope enhancement. Therefore, the response to the short ITI/large ΔF condition might be due to phase resetting, possibly instantaneous sound processing by phase reset. Conversely, the response to a long ITI tended to have a stronger evoked property. The difference between phase reset-like and evoked-component-like properties might reflect whether the stimulus-evoked response was involved in either information processing of continuous perception by ongoing oscillation or transient processing of each tone.

The phase resetting is a possible neural mechanism underlying the build-up effect of streaming. When the phase reset at each tone in the sequence is inadequate due to insufficient stimulus strength, temporal shifts of the phase may accumulate over time, specifically under short ITI conditions [74]. Such an accumulating effect should be involved in the build-up effect of auditory streaming. Another candidate causing the build-up effect is multisecond habituation of single-unit activities in the primary auditory cortex [4]. Because an initiation of action potential depends on synchrony of synaptic inputs as well as strength of inputs, the single-unit activity should be associated with LFP phase modulation underlying synchronized inputs. Thus, our finding in LFP phase modulation is not mutually exclusive with a previous finding at single neuron level in order to explain the build-up effect of streaming.

**Possibility of other neural correlates for streaming.** We cannot exclude a role for amplitude modulation of neural activity as a correlate of auditory stream perception. Under *Integration* conditions—long ITI and small ΔFs— the amplitudes of beta and gamma band oscillations (Fig. 7**B**) were more likely to conform to the psychometric function than ITPC (Fig. 8**B**). In addition, there are other possible candidates of the neural correlates of auditory streaming, such as auditory mismatch negativity [75]–[77], object-related negativity [77], [78], stimulus-specific, multiple-time-scale adaptations [79], and stimulus-induced but non-stimulus-locked concerted activities by neuronal ensemble across auditory cortical fields [80], [81]. Furthermore, interactions between cortical and subcortical activities via cortico-fugal connections should be also taken into account [29].
