# Correction: Stimulus Phase Locking of Cortical Oscillation for Auditory Stream Segregation in Rats

**DOI:** 10.1371/journal.pone.0146206

**Published:** 2015-12-29

**Authors:** Takahiro Noda, Ryohei Kanzaki, Hirokazu Takahashi

There is an error in the second sentence of the second paragraph of the “Behavioral experiment” subsection of the Results. The correct sentence is: Statistically, the poking rate at ΔF = 12 was significantly larger than those at ΔF = 2 and 6 (one-way ANOVA, *F*(2,9) = 4.38, *p* = 0.0470; *post hoc* one-sided *t*-test p = 0.0113 and 0.0265).
